# Practice of the training program for ophthalmic specialist nurses in Zhejiang Province of China

**DOI:** 10.1186/s12912-023-01271-3

**Published:** 2023-04-13

**Authors:** Dandan Jiang, Huarong Chen, Mengyue Zhang, Saijin Zhang, Jingjing Xu, Yanyan Chen, Yinghui Shi

**Affiliations:** 1 National Clinical Research Center for Ocular Diseases, Eye Hospital, Wenzhou Medical University, 270 West Xueyuan Road, 325027 Wenzhou, Zhejiang China; 2 School of Ophthalmology and Optometry, Wenzhou Medical University, 270 West Xueyuan Road, Wenzhou, 325027 Zhejiang China

**Keywords:** China, Ophthalmic specialist nurse, Education, Training

## Abstract

**Aims:**

To explore the effects of training programs for ophthalmic specialist nurses in Zhejiang Province of China.

**Methods:**

The training program included one month of theoretical training and three months of practical clinical training. The Two-Tutor system was used in training. The training contents were mainly set up around four modules: specialty knowledge and clinical skills, management, clinical teaching, and nursing research. We used theoretical examination, clinical practice assessment and trainee evaluation to assess the effectiveness of the training program. Before and after the training, the trainees’ core competence was assessed by a homemade questionnaire.

**Results:**

In total, 48 trainees from 7 provinces (municipalities) in China participated in the training program. All trainees passed theoretical and clinical practice examinations and trainee evaluations. Their core competencies were significantly improved after training (p < 0.05).

**Conclusion:**

This training program for ophthalmic specialist nurses is scientific and effective in improving nurses’ ability to provide ophthalmic specialist nursing care.

## Introduction

China has one of the highest rates of serious blindness and vision impairment in the world. The age-standardized prevalence of moderate vision impairment is 12.17%, much higher than the global average of 1.48% [1]. The high levels of eye disease have led to a huge demand for eye care in China, and more ophthalmic specialist nurses are needed to provide specialist eye care, which is in contradiction with the limited supply of ophthalmic specialist nurses.

With the development of ophthalmology, the demand for highly skilled and well-trained ophthalmic nurses is increasing. Ophthalmic nurses need to have profound theoretical knowledge and clinical skills, along with scientific research abilities so they can grasp the development directions of this field. This drives ophthalmic nurses to become highly specialized.

The document “National Nursing Career Development Plan (2016–2020)“(“National Nursing Career Development Plan (2016–2020),“ 2017) [2] issued by the National Health Commission stated that the number of specialist nurses should be increased and the skills of specialist nurses should be improved. Advanced practice nurses in ophthalmology have been widely used in ophthalmic emergency and subspecialty fields internationally. Meanwhile, demand for ophthalmic specialist nurses are high in China, there should be at least one specialist ophthalmic nurse in each eye ward.

Ophthalmic specialist nurses were trained and certified in the UK in the 1990s [3]. The training program integrated schooling with in-service education [4], focused on ophthalmic specialist knowledge and clinical skills [5], and set up specialist orientation and grade divisions in different areas. The ophthalmic specialist nurses were able to run ophthalmology outpatient clinics independently and undertake some teaching and research work in the UK. In contrast, ophthalmic specialist nurses’ training was mainly in-service training within medical institutions in Africa [6, 7].

Ophthalmic nursing education is not a focus of study in schooling in China, nor is it provided as specialized training when they start working in the hospital. Many ophthalmic nurses are not able to provide high-quality ophthalmic nursing care because of a lack of good specialty knowledge and clinical skills [8]. Different types of training programs for specialist ophthalmic nurses have been carried out in some provinces and municipalities in China since 2014 [9], which is mainly short-term in-service education relying on medical institutions, mostly for 2–3 months. However, different training bases provide different curricula and lack innovative specialty knowledge.

To explore a training program for ophthalmic specialist nurses and improve the level of specialty nursing in China, the Zhejiang Nursing Association established the Ophthalmic Nursing Professional Committee of the Zhejiang Nursing Association in 2016. Subsequently, the first training of an ophthalmic specialist nurse was carried out in 2020. By August 2021, two sessions were held. The purpose of this training was to build a training program for Chinese ophthalmic specialist nurses. The training program will provide a basis for high-quality training of ophthalmic specialist nurses in China.

## Methods

### Trainees

The nurses willing to participate in the ophthalmic specialist nurse training programs were subjected to strict selection criteria. Recruitment requirements were as follows: ① a registered nurse; ② being engaged in nursing work for more than 8 years with a college degree; ③being engaged in nursing work for more than 5 years with an undergraduate or higher degree; ④ being engaged in eye care for more than 3 years and loving it; ⑤ English reading and writing skills.

### Training bases

The training bases are grade III A hospitals in Zhejiang Province, China. All of them are teaching hospitals. These hospitals are capable of providing precision medicine in ophthalmology and treating complex ophthalmic diseases. In addition to that, various ophthalmic specialist nursing operations, perioperative management and care of ophthalmic diseases and personalized service in ophthalmic surgery are carried out in these hospitals. And, they have abilities for conducting teaching training, continuing education and clinical research.

### Trainers

The trainers consisted of project leaders, discipline leaders, theoretical trainers, clinical practice trainers and research instructors. The theoretical trainers were from the training bases and universities. There were university teachers who had the title of associate professor or above, clinical teachers who had the title of associate chief physicians or above, bachelor or above, nurses-in-charge or above, and senior technicians (engineers). In addition to the intensive classes, we had one-on-one clinical practice trainers and research instructors. The clinical practice trainers had an intermediate level or above, with a bachelor’s degree or above. They had been working for more than 5 years. The research instructors had good research ability and English reading and writing skills and were full-time postgraduates or above.

### Training program

Employing the “1 + 3” full-time learning scheme, the whole training lasted for four months, including one month of theoretical training and three months of clinical practice. We consulted the literature and organized the expert meetings. The outline of the training program for the ophthalmic specialist nurse was formed based on the core competency cultivation objectives of the trainees. The training courses were mainly set up around four modules involving specialty knowledge and clinical skills, management, clinical teaching and nursing research (Table 1, Page20). A two-round Delphi survey was conducted to seek opinions from experts about the training program. The authority coefficient and Kendall’s concordance coefficients of Delphi experts were 0.95 and 0.23. The coefficient of variation of all items of Delphi experts were less than 0.28. The training program is scientific and reliable.

**Table 1:** Training course for ophthalmic specialist nurses in Zhejiang Province

Training modules	Submodules	Training contents	Training time	Training modalities
Specialty knowledge and skills	Theoretical knowledge	Treatment and care of common Ophthalmology&Optometry diseases, chronic disease management, perioperative management, case management ,new practices and technologies in Ophthalmology&Optometry nursing, innovative specialty knowledge in Ophthalmology&Optometry	Weeks 1 ~ 4	Theoretical lectures
	Clinical skills	Ophthalmic nursing skills such as common ophthalmic nursing practices, emergency treatment of eye diseases and care of difficult cases	Weeks 2 ~ 16	Theoretical lectures; Workshops; Operation practice
	Clinical practice	Ophthalmology (ward, outpatient, operating theatre), case management, optometry (three centres) and other departmental rotations	Weeks 5 ~ 16	Clinical practice
Management	Ophthalmic nursing management	Design process and management of ophthalmic day ward, integrated management of ophthalmic hand supply, application of rapid response system in ophthalmic emergencies, ophthalmic nursing humanities and nursing culture construction, the application of diversified health education models in ophthalmology	Weeks 1 ~ 16	Special Lectures Departmental Rotation
Clinical Teaching	Teaching Nursing	Teaching design and implementation of effective teaching, skills of presentation of teaching, lesson plan writing, analysis of excellent lesson plans	Weeks 1 ~ 16	Special lectures Small lectures Specialized teaching
Nursing Research	Theoretical knowledge	Literature search, explanation of the key points and techniques of reading nursing papers, qualitative nursing research and the application of Nvivo software, sample size calculation methods	Weeks 5 ~ 16	Theory lectures Special lectures Specialized teaching
	Research Practice	Writing of case reports, overviews and dissertation proposal		
Social Practices	/	Participate in Little Eye Doctor volunteer activities, science museum science activities, community lectures	Weeks 9 ~ 16	Visiting Practices

This training aimed to develop the four core competencies of ophthalmic nurses: (1) specialty knowledge: mastering and applying the theoretical and operational skills of specialist ophthalmic nursing to solve complex clinical problems based on consolidating basic knowledge and skill operations; (2) nursing management: mastering nursing management skills, improving nursing management ability, and possessing better humanistic care and communication skills; (3) clinical teaching: to be able to undertake nursing teaching in the department and guide junior nurses in their work; (4) nursing research: having a spirit of scientific research innovation and the ability to carry out nursing research projects to enhance the level of nursing research in the department.

(1) Specialty knowledge and clinical skills.

The specialty knowledge and skills were mainly set up around three modules involving theoretical knowledge, clinical skills and clinical practice. Theoretical knowledge training was arranged in weeks 1 to 4, with no less than 160 h. The training format included intensive classes and special lectures for five days a week. The training involved the treatment and care of ophthalmic diseases, management of chronic diseases, perioperative management, new technologies in ophthalmic care, case management, and the innovative specialty knowledge of ophthalmology. Clinical skills training included theoretical teaching, workshops and operational exercises from weeks 2 to 16. The training involved both theoretical training and clinical practice. After the theoretical lectures, operation exercises and practical exercises were conducted. Skills training included lacrimal irrigation, ophthalmic dressing changes and removal of foreign bodies from the conjunctiva under a slit lamp.

The clinical practice period was from weeks 5 to 16. All trainees were arranged to practice in the three training bases and taught one-on-one by trainers. They were trained in ophthalmology outpatient, wards, case management and operating theatres. All trainees completed the clinical training program according to the requirements.

(2) Nursing Management.

The management module covered ophthalmic nursing management. The content covered the design process and management of the day wards, the integrated management of the operating room and central sterile supply department and the application of diversified health education models in ophthalmology. The training period was from weeks 1 to 16, and the training format included special lectures and departmental rotations.

(3) Clinical Teaching.

The module of clinical teaching included teaching design and the implementation of effective teaching, courseware production skills, lesson plan writing, analysis of excellent lesson plans, etc.

The training was mainly set up around three forms involving special lectures, small lectures and one-on-one teaching. After training, the trainees were required to make a teaching plan and courseware with one-on-one guidance from the teacher.

(4) Nursing Research.

Training in scientific research was also provided. Combining theory and practice, trainers gave lectures on nursing research knowledge during the intensive learning. During clinical practice, we organized lectures on topics such as sample size calculation and literature searching. Trainees were required to complete one report of case care, literature reviews and a dissertation proposal. The topic of case care report and literature reviews were chosen by trainees. Trainees should complete a dissertation proposal follow the application form of Zhejiang Medicine and Health Science and Technology Project. The dissertation proposal included background, contents and methods of research. Trainees should search literatures and clinical problems, and conduct standardized research design.

### Training assessment

To assess the effectiveness of the training program, we used different assessment methods for each module. In the theoretical training phase, an examination was carried out at the end of the training. The content mainly involved the important and difficult points in the theoretical training. The total score was 100 points. The specialty skills assessment involved process assessment and project assessment. The process assessment meant that the clinical teacher selected “excellent, good or average” according to the mastery of each item by the trainees during training. The project assessment included operation examination and presentation of teaching. In the operation examination, two assessment items were designated in each period, and the trainees were evaluated and scored in the late stage of clinical practice. The PPT lectures were conducted by the trainees, who chose the content of the lectures, completed the lesson plans and PPT lectures, and conducted the classroom lectures. The comprehensive assessment covered the trainees’ self-assessment and the trainer’s evaluation.

Trainees were required to complete a training achievement report. The training achievement report consisted of five modules: case care, review, lesson plans, presentation of teaching and a dissertation proposal. After completing all of the courses and clinical practice, trainees who had passed the theoretical and practical assessments submitted their training achievement report. In addition, they participated in the trainee evaluation by the Expert Committee for Specialist Nurse Training of Zhejiang Province. The expert committee included eye care specialists, medical and surgical care specialists and clinicians. Trainees need to answer experts’ questions, such as nursing knowledge, scientific research design of the dissertation proposal. Experts will give the result of whether or not to pass the trainee evaluation. After passing the trainee evaluation, trainees returned to their hospitals for a one-year qualification period. After being approved by the Specialist Nurse Management Committee, they would obtain the “Certificate of Qualification for Ophthalmic Specialist Nurse Training in Zhejiang Province” issued by the Health Commission of Zhejiang Province.

### Core competency assessment

Before and after the training, the trainees’ core competence was assessed by a homemade questionnaire. The questionnaire was designed by the Training Management Committee. The questionnaire demonstrated a high internal validity with a Cronbach’s α of 0.978. The questionnaire includes 4 dimensions and 36 items: theoretical knowledge, clinical skills, teaching ability and research ability. Each item is rated using a Likert five-level scoring method. All questions are answered from “complete ignorance” to “complete mastery”, scoring from “1” to “5” points. A higher score represents better mastery.

### Data analysis

Excel was used for data entry, and SPSS 24.0 software was used for statistical analysis of the data. Count data are presented as frequencies and percentages, while measurement data are expressed as the means and standard deviations (SDs). The independent samples t-test was used to compare the core competency of the trainees before and after training.

## Results

### Participants

Zhejiang Nursing Association and three training bases released a notice on website and public platform of WeChat at the early of the year. Forty-eight trainees from 6 provinces across the country participated in the training program, including 47 women and 1 man. The average age of the trainees was 33.69 ± 5.36 years. Three (6.25%) trainees had a college degree, 44 (91.67%) had a bachelor’s degree, and 1 (2.08%) had a master’s degree. The distribution of professional titles among the trainees was as follows: 17 (35.40%) primary title nurses (nurse practitioner), 30 (62.50%) intermediate titles (nurse-in-charge), 1 (2.10%) co-chief nurse and above, 37 (77.08%) chief nurses and 11 (22.92%) in other positions, such as assistant to the chief nurse. In terms of departments, there were 32 (66.70%) from ophthalmology departments and 16 (33.40%) from departments where ophthalmology was combined with other diseases or fields.

### Evaluation assessment

After 4 months of specialist nurse training, all trainees completed the theoretical and practical courses and passed the trainee evaluation. The results showed that the four dimensions were improved significantly after the training, and the differences were statistically significant.

In particular, for the theoretical knowledge dimension, the pretraining score for the case management of chronic diseases in ophthalmology was less than 3. This showed that the trainees were not familiar with this dimension, while after the training, the mean score for all entries was more than 3 (Table 2, Page22).

**Table 2:** Comparison of theoretical knowledge dimensions before and after training (n = 48)

Item	Before training(Score)	After training(Score)	t	P
Treatment and care of common ophthalmic diseases	3.83 ± 0.81	4.15 ± 0.65	-2.086	0.040
Treatment and care of common optometric diseases	3.35 ± 1.02	3.83 ± 0.79	-2.583	0.011
Ophthalmic examination care coordination and interpretation of results	3.13 ± 1.00	3.79 ± 0.71	-3.754	< 0.001
New process in the treatment and care of common ophthalmic diseases	3.46 ± 0.92	3.92 ± 0.74	-2.688	0.008
Case management of ophthalmic chronic diseases	2.65 ± 1.18	3.56 ± 0.71	-4.621	< 0.001
Information-based nursing management	3.00 ± 1.05	3.65 ± 0.70	-3.543	0.001
Process and management of the ophthalmic day ward	3.54 ± 0.99	4.10 ± 0.72	-3.184	0.002
Total	22.96 ± 5.78	27.00 ± 4.11	-3.953	< 0.001

In terms of clinical skills, before the training, the scores of conjunctival sac irrigation, lacrimal duct irrigation and ocular dressing change were better than 4. There was no statistically significant difference between the scores before and after the training. However, trainees were not familiar with pseudomembrane removal of the palpebral conjunctiva, conjunctival calculus removal, corneal conjunctival dissection, ocular skin dissection or the use of amblyopia training devices. After the training, the average scores on all skills were higher than 3 (Table 3, Page23).

**Table 3:** Comparison before and after training in operational skills (n = 48)

Item	Before training(Score)	After training(Score)	t	P
Subconjunctival injection	3.10 ± 1.28	3.69 ± 0.97	-2.521	0.013
Subcutaneous injection of superficial temporal artery	3.60 ± 1.38	4.10 ± 0.90	-2.099	0.038
Conjunctival sac irrigation	4.40 ± 0.92	4.52 ± 0.74	-0.734	0.465
Pseudomembrane removal of palpebral conjunctiva	2.69 ± 1.17	3.25 ± 0.98	-2.555	0.012
Conjunctival calculus removal	2.88 ± 1.23	3.56 ± 0.87	-3.156	0.002
Keratoconjunctival foreign body removal	2.75 ± 1.19	3.40 ± 0.84	-3.060	0.003
Lacrimal duct irrigation	4.29 ± 1.07	4.65 ± 0.64	-1.970	0.052
Intra-Ocular pressure measurement (Goldmann applanation tonometry)	3.42 ± 1.44	4.08 ± 0.94	-2.683	0.009
Ocular dressing change	4.13 ± 0.98	4.40 ± 0.79	-1.488	0.140
Ocular skin stitch removal	2.98 ± 1.19	3.65 ± 0.86	-3.136	0.002
Cornea stitch removal	2.67 ± 1.10	3.40 ± 0.84	-3.648	< 0.001
Hot compresses for the eyes	3.79 ± 1.05	4.38 ± 0.79	-3.075	0.003
Meibomian gland massage	3.27 ± 1.27	4.06 ± 0.81	-3.647	< 0.001
Meibomian gland (stye) incision	2.71 ± 1.15	3.33 ± 0.91	-2.960	0.004
First aid for the top 10 ophthalmic emergencies	3.46 ± 0.92	4.02 ± 0.81	-3.173	0.002
Ophthalmic surgical care coordination techniques	2.88 ± 1.21	3.71 ± 0.82	-3.935	< 0.001
Corneal topography measurement	2.65 ± 1.02	3.31 ± 0.83	-3.512	0.001
Use of amblyopia training devices	2.48 ± 0.99	3.17 ± 0.78	-3.780	< 0.001
Life and rehabilitation skills for people with low vision	2.54 ± 1.03	3.38 ± 0.76	-4.506	< 0.001
Total	60.67 ± 16.30	72.04 ± 11.28	-3.975	< 0.001

For clinical teaching, the trainees’ mean scores of all items were higher than 3 points before and after the training. The scores of all items improved after the training, with statistically significant differences (p < 0.05) (Table 4, Page24).

**Table 4:** Comparison before and after training in teaching skills (n = 48)

Item	Before training(Score)	After training(Score)	t	P
Design and application of nursing teaching	3.35 ± 0.91	3.96 ± 0.71	-3.618	< 0.001
Skills of presentation of teaching	3.65 ± 0.76	4.04 ± 0.71	-2.635	0.010
Writing and design of lesson plan	3.29 ± 0.94	3.96 ± 0.71	-3.903	< 0.001
Skills in new forms of delivery such as the flipped classroom	3.04 ± 1.05	3.85 ± 0.80	-4.264	< 0.001
Total	13.33 ± 3.35	15.81 ± 2.78	-3.947	< 0.001

For the nursing research, before the training, the average scores of scientific research design, data analysis, qualitative research, research methods for evidence-based care and writing a dissertation proposal were all less than 3. After the training, the scores for each item improved to more than 3 (Table 5, Page24).

**Table 5:** Comparison before and after training in research skills (n = 48)

Item	Before training(Score)	After training(Score)	t	P
Literature search	3.15 ± 0.95	3.92 ± 0.77	-4.387	< 0.001
Research design	2.90 ± 1.04	3.71 ± 0.80	-4.304	< 0.001
Methods of common data analysis	2.73 ± 1.05	3.46 ± 0.77	-3.887	< 0.001
Writing a standard academic paper	3.04 ± 1.03	3.75 ± 0.69	-3.995	< 0.001
Research methods for qualitative research and evidence-based care	2.75 ± 1.04	3.31 ± 0.75	-3.039	0.003
Dissertation proposal	2.79 ± 1.11	3.81 ± 0.64	-5.517	< 0.001
Total	17.35 ± 5.89	21.96 ± 3.85	-4.532	< 0.001

## Discussion

New technologies and methods of ophthalmic treatment are constantly emerging, posing new challenges to ophthalmic clinical nursing work. At present, ophthalmic nurses in domestic hospitals have varying qualifications and weak basic theoretical knowledge. In nursing work, they rely on simple nursing operations, mechanical execution of medical advice, and low levels of care in difficult cases to help care for patients, which have not yet reached the standard of specialist nursing[10, 11]. The professional value of specialist nurses lies in their ability to solve difficult and complex problems [12]. However, this requires nurses with specialized theoretical knowledge and skills and extensive clinical experience in ophthalmic nursing.

However, the development of training programs for ophthalmic specialist nurses is facing a series of questions, and the process of development varies from country to country. Although the training system of ophthalmic specialist nurses is well-developed in the UK, more than 50% of ophthalmic specialist nurses reported that they could not obtain enough learning resources. They had no chance or time to participate in such training, which was the main barrier to further education. Therefore, some scholars have proposed a curriculum based on the Ophthalmic Common Clinical Competency Framework to improve the clinical skills and knowledge of ophthalmic nurses [13].

There is no uniform standard for the traditional training of ophthalmic nurses in China. They only receive a few days of training on ophthalmic knowledge upon entry, and it is impossible to conduct systematic and in-depth learning. Different scholars have different views on the training focus for ophthalmic specialist nurses. Yang et al. [9]emphasized the training of specialized knowledge and clinical skills, while Zhang et al. [14] believed that in addition to this, clinical management and scientific research should be strengthened in the training contents.

Therefore, it is important to improve the effectiveness of the training program of ophthalmic specialist nurses by properly arranging the training time and content. This training program adopted the “1 + 3” model involving 1 month of theoretical training and 3 months of clinical practice, which provided trainees with systematic and comprehensive specialist knowledge. The theoretical training included basic theoretical knowledge and frontier research advances, using multimodal training methods such as theoretical lectures and workshops. After the theoretical study, clinical practice was carried out. The clinical practice included general wards (corneal specialist, fundus specialist, glaucoma specialist, etc.), day wards, eye outpatient (treatment rooms, TCM treatment areas), optometry, operating theatres, etc. The subspecialties of ophthalmology were subdivided to allow trainees to enter immersive learning, improving the level of care for difficult cases in ophthalmology. The training program provides specialty knowledge and clinical skills. They will be able to attend the meeting held every year by Zhejiang Nursing Association to keep the specialists up-to-date after they graduate.

Specialist nurses are not only advanced practitioners of clinical nursing; their extensive clinical experience and critical thinking also allow them to play an important role in nursing teaching and research innovation. In this study, a survey of the trainees’ purpose for attending specialist nurse training found that the top was to improve research skills (45, 93.80%), followed by improving ophthalmic specialist operational skills (39, 81.30%) and learning about difficult eye diseases (37, 77.10%). In the training system for ophthalmic specialist nurses constructed by Zhang et al. [14], ophthalmic teaching management and scientific research were also incorporated into the clinical practice indicators and assessment indicators. The role definition of ophthalmic specialist nurses was also further refined. However, nurses mainly have college and undergraduate degrees [15] and only a weak foundation in scientific research and teaching in China.

Therefore, the Two-Tutor system was used in training. The clinical trainers were all departmental backbones or head nurses with rich clinical teaching experience. The trainers had some experience in the lesson plan and courseware design, providing guidance for the trainees to produce standardized lesson plans and courseware. The research trainers were all full-time employees with master’s degrees who had good research skills and English skills proficiency, and they had experience in writing theses and using research tools. Thus, the research trainers had the ability to guide students in writing standardized opening reports and reviews. Trainees also required to have English reading and writing skills. On the basis of the Two-Tutors system, our training program provided thematic training on teaching and scientific research to promote the overall improvement of the teaching and research abilities of the trainees. During the one-month theoretical training, intensive lectures were carried out. During clinical practice, relevant experts were invited to conduct weekly training, which included literature searching, research statistics and thesis selection.

New theories, techniques and methods of ophthalmic nursing have been widely promoted and applied in clinical practice, while the scope of ophthalmic specialist nursing has also been expanded and extended. The practice of ophthalmic day wards is becoming increasingly mature. The short, flat and rapid nature of ophthalmic surgery poses a challenge to day ward nursing. Continuity of nursing care has increasingly become the focus of day ward nursing; patients with chronic eye diseases such as glaucoma and uveitis need to maintain good habits and have regular check-ups over time. Case management is extremely important for patients with chronic eye conditions to recover and control their ocular diseases; the “Internet + Nursing services” set higher standards for nursing staff, requiring them to have certain qualifications before they are employed. As a result, ophthalmic specialist nurses are not only required for nursing care in the hospital, but the scope of nursing care extends from the hospital to the community and home. The training program included diversified health education models in ophthalmology, case management and “Internet + Nursing services” to enhance the specialist nurses’ ability of education and early diagnosis competencies in the community. In addition, trainees were able to participate in community activities such as visiting a science museum and a “Little Eye Doctor” to enhance the specialist nurses’ ability to serve the community.

## Conclusion

The training program for ophthalmic specialist nurses in China is still under active exploration and testing. The training program for ophthalmic specialist nurses aims to cultivate clinical nurses who can solve difficult clinical nursing problems and have both teaching and research skills. This training program is reasonably designed, with systematic and comprehensive training contents and scientific and rigorous assessment methods, which have certain reference significance. However, the recertification of the training system for specialist nurses has not been perfected in China, so the future development of ophthalmic specialist nurses still needs to be explored.

## Data Availability

The datasets used and analysed during the current study are available from the corresponding authors on reasonable request.
